# Safety and Efficacy of Hepatic Artery Embolization in Treating Solitary Fibrous Tumor Metastatic to the Liver

**DOI:** 10.1155/2019/3060658

**Published:** 2019-09-03

**Authors:** Sara Velayati, Joseph P. Erinjeri, Lynn A. Brody, Etay Ziv, Franz E. Boas, Karen T. Brown, Anne M. Covey, George I. Getrajdman, Stephen B. Solomon, Peter T. Kingham, William D. Tap, William R. Jarnagin, Hooman Yarmohammadi

**Affiliations:** ^1^Department of Interventional Radiology, Memorial Sloan Kettering Cancer Center, New York, NY, USA; ^2^Department of Surgery, Memorial Sloan Kettering Cancer Center, New York, NY, USA; ^3^Department of Medicine, Weill Cornell Medicine, New York, NY, USA

## Abstract

The aim of this study was to evaluate safety and survival following hepatic artery embolization (HAE) for metastatic solitary fibrous tumor (SFT) in the liver. All patients with SFT metastatic to liver treated with HAE were retrospectively analyzed. Tumor response was evaluated using mRECIST. Objective response, overall survival (OS), and progression-free survival (PFS) were evaluated using Kaplan–Meier and multivariate Cox proportional hazard ratio. Adverse events were graded according to the National Cancer Institute Common Terminology Criteria for Adverse Events, version 5.0. Twelve patients (6 males and 6 females, mean age: 42.5 ± 13 years; 24–65) were treated with 33 embolizations. Anatomical sites of origin for SFT were the head and neck (*n* = 6; 50%), pelvis (*n* = 2), pleura (*n* = 2), retroperitoneal (*n* = 1), and thigh (*n* = 1). The median follow-up from first HAE was 4.5 years (3–7.9). 84% of the patients showed objective response [42% complete response (CR) plus 42% partial response (PR)] to HAE by mRECIST (95% CI, 60–99%). Patients with CR to HAE had significantly higher OS compared to others (*p* < 0.02). The postembolization median OS was 4 years (95% CI, 2.3–5.2), and mean PFS, for intra- or extrahepatic progression of disease, was 6 months (95%, CI, 3.2–7.1). One patient developed pneumonia/sepsis and died 27 days postembolization, possibly not directly related to embolization. No grade III or IV adverse events were identified in the remaining patients. In conclusion, HAE for metastatic liver SFT is a relatively safe treatment option with high response rate and should be considered as a treatment option for metastatic liver SFT. In our cohort of patients with metastatic SFT to the liver, we observed a median OS of 4 years following HAE. Further studies are needed to confirm the efficacy of HAE.

## 1. Introduction

Solitary fibrous tumor (SFT), originally called hemangiopericytoma, was initially described in 1942 by Stout and Murray as a soft tissue neoplasm arising from pericytes of Zimmerman [1]. This entity was reclassified in 2006 as solitary fibrous tumor [2]. It affects adults aged 20–70 years (median age of 40s), and the most common sites for primary involvement are the extremities (axilla and thigh), pelvis/retroperitoneum, head and neck, and lung/pleura [3, 4].

Surgical resection of the primary tumor is the treatment of choice and provides a 10-year overall survival (OS) of 58% [5]. However, once the patient develops metastatic disease, the OS significantly decreases with 5-year overall survival of 11% [5].

The liver is one of the most common metastatic sites [6]. Unfortunately, chemotherapy and radiation therapy provide limited responses. The aim of this study was to evaluate the safety and efficacy of hepatic artery embolization (HAE) in patients with metastatic liver SFT.

## 2. Materials and Methods

Under an institutional review board waiver, a retrospective review of all patients who underwent HAE between 2003 and 2018 was performed to identify patients treated for metastatic SFT to the liver. Clinical information and follow-up data were obtained from the electronic medical record. The histological diagnosis of SFT was established according to the World Health Organization Classification of Tumors. The decision to perform HAE was made by a multidisciplinary team including surgeons, oncologists, and interventional radiologists.

### 2.1. Patient Population

Patient demographics, clinical presentation, disease status at diagnosis, liver tumor size, and number, site of primary tumor/metastases, initial treatment, type of treatment, and outcome were obtained from review of the electronic medical record. All patients underwent routine physical examinations, laboratory tests, and cross-sectional imaging studies including imaging contrast-enhanced computed tomography (CT) or magnetic resonance imaging (MRI) before HAE.

### 2.2. Hepatic Artery Embolization (HAE)

HAE was performed using our previously described method as selectively as possible using microcatheters [7]. Embolization was performed using Embosphere® microspheres (40–120 or 100–300 *μ*m; Merit Medical, South Jordan, UT), Bead Block® (100–300 *μ*m; BTG International Ltd, London, UK), or Embozene™ microspheres (100 *μ*m; Boston Scientific, Marlborough, MA) depending on users' preference. The endpoint of embolization was complete stasis defined as the absence of antegrade flow in the treated vessel without washout, five cardiac beats after injecting the contrast [8]. Completion of treatment was defined as the time that embolization of all hepatic tumors was complete. In patients with disease limited enough to allow treatment of all tumors in one setting, this was the time of the first HAE; in patients with bilobar disease not amenable to treatment in one session, this was defined as the time that the second embolization was completed.

### 2.3. Follow-Up Evaluation

Patients were followed at 4–6 weeks after each embolization with routine physical examinations, laboratory examinations, and cross-sectional imaging studies. Contrast-enhanced multiphase CT or MR imaging was performed at 1 and 3 months after HAE and subsequently every 3–6 months after the procedure.

### 2.4. Response Analysis

Initial therapeutic response was evaluated between 1 and 3 months postembolization, using modified RECIST (mRECIST) [9]. OS and PFS were calculated for surgery and HAE. OS for surgery was calculated from the date of tissue diagnosis either surgical or by biopsy to the date of death or to the date of the last follow-up for patients that were alive at the time of preparation of the manuscript. For HAE, OS was calculated from the time of the first HAE to the date of death or to the date of the last follow-up for the patients that were alive at the time of preparing this manuscript. PFS for surgery was defined from the date of resection to the date of recurrence of disease or progression of disease in patients that presented with metastatic disease. PFS for HAE was defined from the date of completion of treatment of the HAE to the date of extrahepatic or hepatic disease progression, date of death, or date of the last follow-up for patients without disease progression. In patients that underwent multiple HAEs, the PFS and OS were only calculated for the first completed HAE treatment. OS was not calculated for the chemotherapy regimens. PFS was calculated for each chemotherapy regimen separately. PFS for chemotherapy regimens was defined from the date of initiation of the treatment regimen to the date of extrahepatic or hepatic disease progression, date of death, or date of the last follow-up for patients without disease progression.

### 2.5. Statistical Analysis

Initial therapeutic responses, OS, PFS after surgery, chemotherapy, and HAE were evaluated using Kaplan–Meier and multivariate Cox proportional hazard ratio. All statistical analyses were performed with software (IBM SPSS statistics version 25; EXCEL version 1803). A *p* value < 0.05 was considered statistically significant.

## 3. Results

### 3.1. Demographic and Tumor Characteristics

Twelve patients (6 males and 6 females) were treated with 33 (mean = 2.75; range 1–9) embolizations. Demographic characteristics of these patients are demonstrated in Table 1. Mean age was 42.5 ± 13 years (range: 24–65 years).

The primary anatomical site of SFT was the head and neck in 6 patients (50%), pelvic region in 2 (16.7%), pleural region in 2 (16.7%), retroperitoneal in 1 patient (8.3%), and thigh in 1 patient (8.3%). Four patients in the head and neck group had meningeal SFT. The majority of patients (10/12, 83.3%) presented with localized disease at the time of initial diagnosis. Only one patient presented with liver metastasis at the time of initial diagnosis. In the remaining 11 patients, the interval between the diagnosis of the primary tumor to that of liver metastasis ranged from 2 to 20 years (mean of 8.5 ± 6.8 years). Five patients had solitary liver lesions when they presented with hepatic metastases. Mean tumor size at the time they were first embolized was 7.3 ± 4.1 cm (range: 2.4–15.5 cm).

### 3.2. Treatments: Surgery, Chemotherapy, and Locoregional Therapy

Surgery was feasible as first-line treatment in all 12 patients. However, one patient with presacral tumor refused surgery to avoid a possible colostomy. The patient had liver metastasis at the time of presentation with presacral tumor. The presacral tumor was radiated, and the liver disease embolized with the hope of ultimately resecting the tumor. However, the pelvic tumor did not shrink enough to allow for a limited resection. The remaining 11 patients were treated with surgical resection at the time of diagnosis.

Ten (83%) patients received chemotherapy including doxorubicin, ifosfamide, dacarbazine, temazolomide plus bevacizumab, sunitinib, sorafenib, brivanib, and pazopanib.  Table 2 summarizes the treatments each patient received, including surgery, radiation therapy, chemotherapy, and locoregional therapies.

In all 12 patients, the liver was the main site of metastasis. Three patients received HAE as first-line therapy, 4 patients as second-line therapy (after one line of chemotherapy had failed), 2 patients received HAE as third-line treatment (after 2 lines of chemotherapy had failed), and 3 patients was treated with HAE as the 4^th^ line of treatment (after 3 lines of chemotherapy had failed) (Table 2). In 5 patients, percutaneous ablation was also performed, two of which embolization, and ablation was performed at the same time. Three patients were treated with radioembolization in addition to hepatic artery embolization, two prior to embolization and one after HAE was performed. Three patients were treated with liver resection, two prior to embolization and one after HAE was performed.

### 3.3. Adverse Events

One patient developed bacteremia and died 27 days postembolization (1/33 embolization: 3%). This patient was a 46 y/o male who initially presented with meningeal SFT. He developed bone metastasis 14 years later and a pancreatic metastasis shortly thereafter. He underwent a Whipple for his pancreatic tumor. He developed liver metastasis 16 years after initial resection and later developed pelvic peritoneal metastasis. The patient had been treated with liver ablation, radioembolization, 8 prior HAEs, and multiple regimens of chemotherapy. His 9^th^ HAE was uneventful until day 2 postembolization when he developed hemorrhagic shock secondary to bleeding from a pelvic implant, unrelated to his liver embolization. This resulted in intensive care unit admission and was later complicated by bacteremia and pneumonia. He recovered and was discharged home but died shortly after discharge; specific cause of death was unclear. Despite prior Whipple, there was no imaging evidence to suggest that any of the embolized tumor had become infected. No grade III or IV adverse events were reported in the remaining patients. The median follow-up from initial diagnosis was 13.2 years (range, 3 to 31 years). The median follow-up from the first HAE was 4.5 years (range, 3–7.9 years).

### 3.4. Response, PFS, and OS

**Figure 1:**
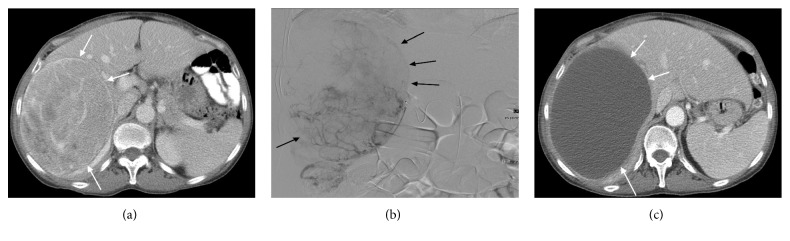
(a) CT scan of a patient with metastatic SFT in the liver prior to embolization demonstrating a 15.5 cm hypervascular mass in the right lobe of the liver (white arrows). (b) Fluoroscopic image of the same patient during hepatic artery embolization demonstrating a large hypervascular tumor in the right lobe of the liver (black arrows). (c) CT scan of the same patient one-month postembolization showing complete response as per mRECIST with no evidence of the residual viable tumor (white arrows).

Complete response (CR) was seen in 42% of the patients with partial response (PR) in another 42%. Therefore, initial response rate (CR + PR) to HAE was 84% (95% CI, 60–99%). Figures 1(a)–1(c) demonstrate an example of complete response after HAE. OS in patients that demonstrated CR was 71.6 months and was significantly higher compared to PR and stable disease (27.2 and 31.5 months, respectively; *p* < 0.02).

**Figure 2:**
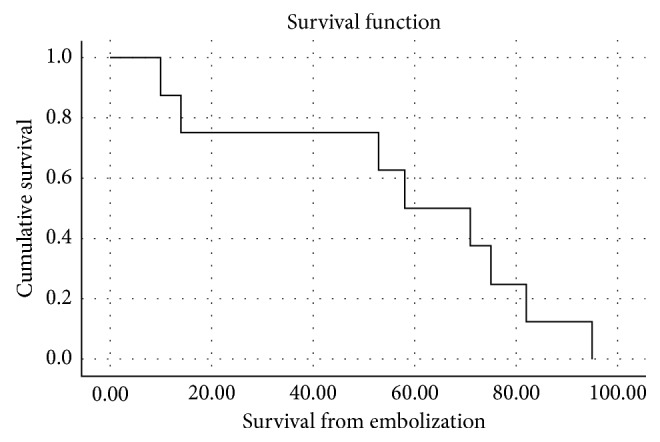
Overall survival after hepatic artery embolization.

The five- and ten-year OS rates from diagnosis were 100% and 63% (95% CI, 25–89), respectively. The postembolization median OS was 4 years (95% CI, 2.3–5.2), and mean PFS was 6 months (95%, CI, 3.2–7.1).  Figure 2 shows Kaplan–Meier graph of OS in patients treated with HAE.

## 4. Discussion

Despite good overall prognosis for SFT, local recurrence or metastases occur in 15–20% of patients, in some cases more than 20 years after the initial management [10]. Most common location of metastases is the lung, liver, and bone [3, 10]. Unfortunately, management of metastatic disease, particularly liver metastases, is challenging, and there is no clear universally accepted guideline. Reported treatment options are resection or metastatectomy, when feasible, and there are various chemotherapy regimens [3, 10, 11].

Radiation therapy has been used as primary therapy or adjuvant therapy in SFT involving the head and neck (intracranial and extracranial), chest, and thoracic, including in 4 of our patients with meningeal SFT [12–14]. Van Houdt et al. reviewed 81 patients with primary and metastatic SFT in which 19 patients were treated with adjuvant radiotherapy. Most of these patients had tumors localized in the head and neck region (*n* = 9; 45%) with unfavorable tumor characteristics such as high mitosis rate and large sizes. It was not clear from the study how many patients treated had metastatic liver SFT. They concluded that radiotherapy offered no significant beneficial effect [13]. Recent study on 40 patients with SFT treated with radiation therapy suggested clinically meaningful benefits with both definitive and palliative intent [14]. However, it was not clear how many of the treated patients had metastatic liver disease. Therefore, there is no defined role for radiation therapy in treating metastatic SFT to the liver.

There is no standard chemotherapy treatment for SFT patients with liver metastasis. Park et al. retrospectively analyzed 21 patients treated with 25 different cytotoxic chemotherapy regimens including doxorubicin-based regimens (*n* = 15), gemcitabine-based therapy (*n* = 5), and paclitaxel (*n* = 5) [15]. The response rate was poor with objective response of 0% and median PFS of 4.6 months [15]. In our study, 3 patients were treated with cytotoxic chemotherapy including doxorubicin, dacarbazine, and ifosfamide with similar poor response rates. With that in mind and considering the fact that SFT is typically a richly vascularized tumor, treatment with antiangiogenic agents was investigated [16–21]. These include interferon-alpha, antivascular endothelial growth factor (VEGF) drugs, and tyrosine kinase inhibitors (TKIs). Park et al. treated 14 patients with temozolomide (150 mg/m^2^ orally on days 1–7 and days 15–21) plus bevacizumab (5 mg/kg intravenously on days 8 and 22, repeated at 28-day intervals). The estimated median PFS was 9.7 months [18]. Five of our patients were treated with temozolomide + bevacizumab regimen with similar PFS to Park et al. In regard to TKI agents, case reports and small series have reported similar results with imatinib, sorafenib, and sunitinib in patients with metastatic SFT [19–22]. In a recent phase II study, sorafenib was used in treatment of soft tissue sarcoma, 5 of which were metastatic SFT, and the authors reported stable disease for 5 months by RECIST [22]. In our study, 1 patient was treated with sorafenib and 7 with sunitinib. PFS was 6.6 months which is similar to previously reported data. In a phase II study by European organization for research and treatment of cancer-soft tissue and bone sarcoma group, pazopanib, a multikinase angiogenesis inhibitor, was used to treat patients with relapsed or refractory advanced soft tissue sarcoma. They were able to conclude that pazopanib was relatively well tolerated and was able to prolong OS and PFS in the heterogeneous soft tissue sarcomas in the study [23]. In our study, 3 patients received pazopanib. These patients' disease remained stable for 5, 7, and 11 months (mean = 7.7 months) which is similar to those previously reported.

Hepatic artery embolization occludes the terminal arterial supply to the tumor causing hypoxia and ischemia and has been used to effectively treat vascular tumors. Since SFT is a vascular tumor, it is not unreasonable to expect that HAE could effectively provide tumor control. In our study, all 12 patients had hypervascular tumors in the liver both on CT scan and angiography (Figures 1(a) and 1(b)). In a recent study, 11 patients with gastrointestinal stromal tumor (GIST) who were refractory to first-line imatinib and second-line sunitinib were treated with HAE [24]. Takaki et al. reported a mean OS and PFS of 23.8 and 3.4 months. GIST tumors are similar to SFT in that they are both sarcomas and both are vascular tumors. In our study, mean PFS after HAE was 6 months which is slightly higher than HAE in GIST most likely due to the more aggressive nature of GIST compared to SFT. Median OS in the 12 patients treated with HAE after embolization was 4 years which is higher than prior case reports with other treatment modalities. To the best of our knowledge, this is the first report on utilization of HAE as a treatment option for metastatic SFT to the liver, and based on these results, we believe that HAE is a safe and effective treatment option for metastatic liver SFT and should be considered as part of the treatment armamentarium. One of our patients with SFT presented with hypoglycemia as a paraneoplastic syndrome. Her symptoms completely resolved after HAE. Therefore, another benefit of performing HAE is rapid symptomatic relief from hormone producing tumors. These characteristics of HAE have been previously reported by Brown et al. in patients with metastatic neuroendocrine tumor to the liver [25]. HAE was able to control hormonal symptoms in 89% of those patients.

This study has several limitations, including the retrospective design and small number of patients. Future studies with larger number of patients would be useful to further define the role of HAE for SFT metastatic to the liver. All of the study patients with metastatic disease were treated with methods other than HAE, and 4 of the 12 patients treated with HAE remained on TKI treatment after embolization. It is possible that the systemic therapy might have prolonged the OS and PFS.

## 5. Conclusions

In conclusion, HAE is a safe and effective treatment option for metastatic liver SFT and should be considered part of the treatment armamentarium for SFT. Further studies are needed to confirm the efficacy of HAE.

## Figures and Tables

**Table 1:** Demographic characteristics of 12 patients with metastatic liver hemangiopericytoma/solitary fibrous tumor treated with hepatic artery embolization.

Demographic characteristics	Number of patients (%)
Age (yrs) (mean ± SD)	42.5 ± 13 (range: 24–65)
Sex	
Male	6 (50%)
Female	6 (50%)
Origin of primary tumor	
Thigh	1 (8.3%)
Retroperitoneal (lumbar)	1 (8.3%)
Pelvis	2 (16.7%)
Pleural	2 (16.7%)
Head and neck	6 (50%)
Cerebellar	1 (8%)
Posterior auricular	1 (8%)
Meningeal	4 (33%)
Metastasis at the time of presentation	
Yes	2 (17%)
No	10 (83%)
Grade	
High	5 (42%)
Low	3 (25%)
Unknown	4 (33%)
Sign and symptoms	
Local pain or tenderness	4 (44%)
Neurologic	4 (44%)
Unknown	2 (22%)

**Table 2:** Treatment regimen in the 12 patients treated with hepatic artery embolization.

Patient number	Location of primary disease	Treatment for primary disease	ChemoRx	HAE, line of therapy	Other interventions
1	H&N (paraspinal)	Surgery	None	1^st^	Hepatic resection (post-HAE)
2	H&N (brain)	Surgery + radiation	Sunitinib TMZ + Bev	3^rd^	—
3	H&N (brain)	Surgery + radiation	Sorafenib	3^rd^	Hepatic resection (pre-HAE)
4	H&N (meningeal)	Surgery + radiation	None	2^nd^	Hepatic resection (pre-HAE) + ablation
5	H&N (meningeal)	Surgery + radiation	Sunitinib Brivanib TMZ + Bev	4^th^	—
6	H&N (meningeal)	Surgery + radiation	Sunitinib Doxorubicin TMZ + Bev	1^st^	Ablation
7	H&N (meningeal)	Surgery	Doxorubicin Pazopanib TMZ + Bev	1^st^	Ablation + Y-90
8	Pelvis (presacral)	Radiation + HAE + ChemoRx	Sunitinib	2^nd^	Ablation × 2, Y-90
9	Pelvis (presacral)	Surgery + radiation	Ifosfamide Adriamycin Dacarbazine Sunitinib	4^th^	Y-90
10	Pleura	Surgery	Pazopanib	2^nd^	PVE + ablation
11	Pleura	Surgery	Sunitinib Pazopanib TMZ + Bez	4^th^	—
12	Thigh	Surgery	Sunitinib	2^nd^	—

H&N = head and neck; HAE = hepatic artery embolization; ChemoRx = chemotherapy; surgery = surgical resection; Y-90 = radioembolization; TMZ + Bev = temozolomide + bevacizumab; PVE = portal vein embolization.

## Data Availability

The retrospective data used to support the findings of this study have not been made available because of ownership reasons.
